# Annual Address of the President of the Texas Academy of Science*From the Transactions of the Texas Academy of Science.Note.—In the Transactions of the Texas Academy of Science the foregoing address is accompanied by a Bibliography, containing, in addition to the footnotes, 115 references.

**Published:** 1901-01

**Authors:** Henry Winston Harper

**Affiliations:** Associate Professor of Chemistry, and Director of the Chemical Laboratory in the University of Texas, Main Department, Austin, Texas


### TEXAS MEDICAL JOURNAL.

ESTABLISHED JULY, 1885.

PUBLISHED MONTHLY.—SUBSCRIPTION $1.00 A YEAR.

VOL. XVI. AUSTIN, JANUARY, 1901. No. 7.

### Original Contributions.

### ANNUAL ADDRESS OF THE PRESIDENT OF THE TEXAS ACADEMY OF SCIENCE.*

### Some Advances Made in Our Knowledge of Immunity and Protective Inoculation.

BY HENRY WINSTON HARPER, PH. G., M. D., F. C. S.,
Associate Professor of Chemistry, and Director of the Chemical Laboratory in the University of Texas, Main Department, Austin, Texas.

In grateful acknowledgment of the honor done me by the fellows and members of the Texas Academy of Science, it is my purpose this evening to bring before you "Some Advances Made in Our Knowledge of Immunity and Protective Inoculation."

"When we search the history of the development of scientific truth we learn that no new fact or achievement ever stands by itself, no new discovery ever leaps forth in perfect panoply, as Minerva did from the brow of Jove.

"Absolute originality does not exist, and a new discovery is largely the product of what has gone before.

" 'We may be confident that each forward step is not ordered by

*From the Transactions of the Texas Academy of Science.

NOTE.—In the Transactions of the Texas Academy of Science the foregoing address is accompanied by a Bibliography, containing, in addition to the foot-notes, 115 references.

one individual alone, but is also the outcome in a large measure of the labors of others. The history of scientific effort tells us that the past is not something to look back upon with regret—something lost, never to be recalled—but rather as an abiding influence helping us to accomplish yet greater successes.'[1a]

"Again and again we may read in the words of some half-forgotten worthy the outlines of an idea which has shone forth in later days as an acknowledged truth."—*Sir William MacCormac.*[1]

The fact that persons once afflicted with smallpox rarely experienced a second attack of that disease when repeatedly exposed to it, was not only early observed, but made a matter of record by the Chinese long before the beginning of the Christian era. That the disease was contagious had long been a matter of common experience, and the means of protection against its ravages early became an interesting subject for investigation.

The Chinese observed that when the dried and pulverized material from smallpox pustules was blown into the nostrils of persons who had not experienced an attack of the disease, that the disease in persons thus infected underwent a milder course, was accompanied by a lower death rate, and conferred immunity against further attacks of smallpox. This early method of protection against the ravages of the disease became a common custom in China and India; but was later superseded by a more direct method of inoculation—that of introducing beneath the skin the scab of variolus pustules. The Chinese used the dried scab, the ordinary Hindoos the fluid pus, and the Brahmans pus that had been kept in wool for a period of twelve months. The latter is clearly an instance of using attenuated virus.

It should be remembered that smallpox extended westward to Europe during the sixth century, that it reached England toward the close of the ninth century, and at the time of the Crusades became widespread. In 1517 it was carried from Europe to Santo Domingo; reached Mexico in 1520, whence it spread throughout the New World. It was introduced into Iceland in 1707, and to Greenland in 1733.

It should be particularly noted, that in the invasion of new territory the virulence of smallpox at once became greatly intensified—in some instances nearly one-half the population being destroyed by it. Robertson records the death of three million and a half of people in Mexico alone as the result of the invasion of 1520. Again, the dark colored races seem to be more easily infected than Europeans.

The protective method of directly inoculating the pulverized variolus scab beneath the skin slowly traveled westward; so slowly, that it did not reach Western Europe until 1718, when Lady Mary Wortley Montagu introduced the process then in vogue in Constantinople. While the year 1718 marks the introduction of protective inoculation to the aristocracy of England, the practice had come into use among Scotch and Welch peasants at a much earlier date, which probably accounts for the next stage in the evolution of measures of protection against infectious diseases.

Herdsmen and milk-maids in both England and Schleswig-Holstein observed that occasionally on the udder of cows there appeared an eruption resembling smallpox; that this eruption could be communicated to persons in milking; and that persons infected with the cowpox were protected against an invasion of true smallpox. The fact that the notorious Mrs. Palmer, Duchess of Cleveland, was thus protected is evidence sufficient to show that such observations were common as early as 1663. In 1768 Fewster and Sutton in London; 1774, Jesty, a Dorsetshire farmer; 1791, Pless, a Holstein teacher, and May 14, 1796, Jenner, confirmed these observations. It is true that the immortal work of Jenner began as early as the year 1769, for at that time, while a student under John Hunter, he heard a young country-woman, in whose presence the subject of smallpox was mentioned, say: "I can not take that disease, for I have had cowpox." Upon mentioning the subject to his master, Hunter replied: *"Do not think, but try; be patient, be accurate."* Jenner did try; was patient, was accurate; and on May 14, 1796, after years of patient labor, in his "Inquiry into the Causes and Effects of the Variolae Vaccinae,"[2] he experimentally established the following facts:

"(1) That this disease (cowpox) casually communicated to man has the power of rendering him unsusceptible of smallpox.

"(2) That the specific cowpox alone, and not other eruptions affecting the cow, which might be confounded with it, had this protective power.

"(3) That the cowpox might be communicated at will from the cow to man by the hand of the surgeon, whenever the requisite opportunity existed; and

"(4) That the cowpox once ingrafted on the human subject might be continued from individual to individual by successive transmissions, conferring on each the same immunity from smallpox as was enjoyed by the one first infected direct from the cow."

Thus it is seen that Jenner, by inoculating a cow with variolus matter produced in the cow an eruptive disease resembling smallpox, but of a milder type, and that the cultivation of this milder disease in the cow yielded a fixed virus (vaccine) which, transplanted to man gave rise to a still milder eruptive disease (vaccinia) possessing constant characteristics, and conferring upon persons who underwent it immunity against smallpox.

The older methods of inoculation against smallpox were quickly supplanted by the simpler and far safer method of vaccination; and since the introduction of the latter the appalling ravages of smallpox have been relegated to historical literature.

The subsequent development of vaccination is a matter of such general information there is no need of its further discussion here. It is sufficient to say that in the great majority (if not in all) of the cases of successful vaccination immunity against smallpox is conferred for an indefinite period, varying from three years to many years—averaging three to seven years—in some cases for life; and that compulsory vaccination and revaccination offers the safest and surest protection against this loathsome disease.

The pronounced success of vaccination gave great impetus to the investigation of the problem of immunity, and the annals of the nineteenth century contain a voluminous record of the prolonged and patient efforts of a host of brilliant workers whose contributions have at least laid the foundation upon which the solution of the problem may, in the future, be built. The building of this foundation can not be recounted here; but it will be necessary to mention some of the materials of which it is made that the latest progress may be intelligently discussed.

As in the case of smallpox, it had long been a matter of common observation that a number of the acute infectious diseases occur but once in the same individual. Whooping-cough, measles, scarlet fever and yellow fever are notable examples of acute infectious diseases, one attack of which usually confers immunity against subsequent attacks of the same disease. It was also observed that some infectious diseases confer a very evanescent type of immunity, and that others confer no immunity whatever.

From the standpoint of immunity the infectious diseases may be easily divided into three classes:

1. Diseases one attack of which confer immunity against subsequent attacks of the same disease.

2. Diseases one attack of which confer immunity against subsequent attacks of the same disease for only short periods of time.

3. Diseases an attack of which confer no immunity whatever.

It would seem that these facts, coupled with Jenner's discovery of a fundamental and practical method of producing artificial immunity, clearly outlined the path for future workers to follow; but, strange to say, the nineteenth century was well on its way before this important route found many followers.

The failure to fully appreciate Jenner's brilliant discovery, and to apply his method to the study of other infectious diseases, finds an explanation in the hazy theoretical conceptions of the cause and nature of infectious diseases which prevailed during the early part of the century. The investigations of fermentation by Astier, Sette, Franz Schulze, Cagnaird de Latour, Schwann, Fuchs, Remak, Mitscherlich, Helmholtz, and others, did much toward clearing the haziness of that period; but it was the monumental work of Pasteur that "finally established the truth of the view that all processes of fermentation and putrefaction alike are caused by living things, and that in each different fermentation different kinds of microbes are concerned."[3] In the light of newer knowledge this statement needs revision. The investigations of Koch on anthrax soon followed, and then came the growth of pure cultures of several pathogenic bacteria.

"The work of Pasteur and Koch afforded the first basis on which the study of artificial immunity could be again undertaken. The possibility of voluntarily producing a number of the most important infectious diseases of men and animals, and of modifying at will pure cultivations of bacteria, either, according to Jenner's precedent, by passage through the animal body, or otherwise in artificial culture media, laid the foundation on which advancement could proceed. Pasteur himself was the first, after Jenner, to produce an artificial immunity by using an attenuated virus; and he was also able to introduce the procedure to some extent into practice with most beneficial results. Still the theoretical explanation of all these facts lagged far behind their practical effect. The very able investigations of Metschnikoff and his theory of phagocytosis were, to many investigators, inconclusive."[4]

Numerous attempts were made to formulate adequate theoretical explanations of the accumulated facts concerning the phenomena of infectious diseases. The followers of Sydenham looked upon the specific disease itself as an *entity;* while Lotze and Virchow viewed it as a *process.* It was clear that a mechanical or dynamical process could not be a living entity. The physiologists Haller, Rail and Johannes Müller had established this principle for normal life processes, and its extension to abnormal life processes was simple enough. "Whatever be the outside forces that act, the eye perceives only light, and the ear only sound; the glands simply secrete and the muscles contract. It is, therefore, the internal condition of the organism, of its organs, tissues or cells, that alone determines the character of the effect. The impulse that must come from the outside to produce these effects is called the stimulus. Hence, there must exist a fundamental internal organization; that is to say, a predisposition to something external * * *. Disease, then may be regarded as the effect produced by quantitative changes in normal conditions, either when the physiological organization is too feeble or the stimulus too intense."[5] Disease may be viewed as a phenomenon of adaptation.

Against this conception, the parasitic or germ theory developed by Plenciz, Eisenmann, Henle, Davaine, Pasteur, Klebs, F. Cohn, J. Schroter, and Koch, appeared to introduce an entirely new qualitative element. It asserts: "That many diseases are due to the presence and propagation in the system of minute organisms having no part or share in its normal economy."[6]

Another conception is that of Pettenkofer, which holds that the determining cause is to be found in the external conditions which vary according to time and place.

It is not difficult to see that these theories are upholding entities as the cause of disease. While a kernal of truth is to be found in each, they all fail to realize the continuity of causes in the sense of modern exact science. "The true and sufficient cause of any effect is always something internal, something that follows from the kind and amount of initial energy, and from that quality and quantity alone and entirely. * * * It is the absolute thing 'that exists behind all change and remains primordially the same,' as Helmholtz expressed it."[7] Or as the modern physicist would put it: potential energy = cause, kinetic energy = effect; and as a liberating impulse will change potential energy into kinetic energy, so a liberating impulse will change cause into effect.

The cloudiness that characterizes many of the theories that have sought to explain the phenomena of infectious diseases is largely a legacy of Kantism, and is clearly out of place in these days of modern science. It is somewhat strange that "ontological toys" are still to be found in the workshop of some really brilliant investigators of natural phenomena. Nevertheless, they are there—which explains some explanations that do not explain.

The parallelism which subsists between the phenomena of fermentation, infection and immunity suggests the mental route to be traveled if an insight into our problem is to be gained; and for this reason it is necessary to first point out a few facts about

FERMENTATION.

If the phenomena of matter be defined as periodic functions of the atomic and molecular masses which constitute it and the rates of motion of these masses, and the chemical unit be viewed as a "center through which energy manifests itself," then the theories of modern chemistry should supply an explanation of the phenomena of fermentation.

The crucial test of every theory which seeks to explain fermentation is the satisfactory explanation of the following phenomena:

Enzymes appear to be capable of disrupting complex chemical bodies without undergoing any apparent chemical change themselves—that is, they bring about a chemical change in disproportionately large quantities of material. When the newly produced subtances attain a certain concentration the further action of the enzyme is inhibited, but its action is reasserted when the concentration of the zymolytic products is again lowered. Maximum, minimum and optimum temperature and pressure influence these changes. The introduction of certain chemical bodies also exert an accelerating or retarding influence; and phenomena of *selective* action are likewise to be found.

Many hypotheses have been submitted. Very ingenious explanations of some of the phases of fermentation are to be found in them; but under the searching light of completer knowledge their incompleteness is sooner or later developed. Many of the modern theories are little else than translations of the earlier hypotheses into terms of modern scientific terminology, so that the later literature is laden with modern extensions of the catalytic theory of Berzelius, Beal's bioplastic theory, Justus von Liebig's physical theory, the germ theory, etc.

Interesting and enlightening as some of these theories are their full consideration is not within the purpose of this address, the limits of which will permit only a brief and incomplete review of some of the more modern conceptions of fermentation, to which attention is now asked.

The more recent investigations of the organized and unorganized (soluble) ferments has dealt a severe blow to the vitalistic theory of fermentation. Hansen's admirable biological researches upon the yeasts, followed by the important investigations of Buchner,

A. Croft Hill, Emil Fischer, and many others, brought to light many interesting hitherto hidden facts; and it now seems clear that all the phenomena of fermentation may be explained from a purely chemical basis. The so-called organized ferments appear to be "active proteids," and the unorganized ferments, or enzymes, are mostly proteid-like bodies presenting great differences in the complexity of their chemical structure.

Hueppe[8] looks upon "active proteid" as "a kind of intermediate stage between lifeless 'nutritional' proteid and living cells"; that it "appears like an anhydride of dead proteid," inasmuch as hydration converts it into an inactive form. Investigations of Bokorny and Loew[9] demonstrated the existence of active proteid in many plants. Loew[10] speaks of it as reserve protein matter of a highly labile nature, and that it differs from all other reserve proteins. He called it proto-protein, and suggested that it is the "material which, by being converted into organized nucleo-proteids, forms living matter." Protein comprises all kinds of albuminous matter, while proteid is used to designate complex compounds of proteins, such as nucleins, haemaglobin, etc. *Labile* chemical compounds are unstable bodies which easily undergo chemical change. Labile atoms or groups of atoms are atoms or groups of atoms which readily migrate from a center of instability to one of stability. When the migration is intramolecular a stereoisomeric compound is the product of the change; when the migration is extra or intermolecular disruption of the molecule takes place. Loew[11] points out the necessity of distinguishing between "potentially labile and kinetically labile compounds; in other words, between static labile and dynamic labile"—using the potential chemical energy in the sense of intramolecular chemical energy. Nitroglycerole and certain other explosive organic compounds represent the potential type, while examples of the kinetic are found in the aldehydes and ketones.

The energy stored in a labile compound is beautifully illustrated in the explosion of the trinitrate of glyceryl — $CH_2(ONO_2).CH(ONO_2).CH_2(ONO_2)$, which, when heated to 257° C., or when struck, explodes with great violence. The products of the decomposition are represented by the equation:

$$4C_3H_5.(NO_3)_3 = 12CO_2+10H_2O+6N_2+O_2.$$

At the temperature of the explosion all these products are gases, and at atmospheric pressure will now occupy the space of about

10,400 litres, having expanded about 18,324 times its original volume.

Another instance is that of mercury fulminate $[(C:N.O)_2 Hg_2 + {}^1/_2 H_2O]$, which develops a pressure of 43,000 atmospheres by detonating in its own volume.

Chemical changes partially or completely destroy the statically labile compounds, while the dynamically labile compounds readily pass into isomeric or polymeric compounds as a result of atomic migrations, or by polymerization. The classic illustration usually given of the production of an isomeric compound produced by atomic migration is Woehler's famous discovery: the transformation of ammonium cyanate into urea, which he accomplished in 1828, by evaporating an aqueous solution of ammonium isocyanate. The transformation is represented by the equation:

$$O:C:N.NH_4 \longrightarrow O:C\begin{matrix} \diagup NH_2 \\ \diagdown NH_2 \end{matrix}.$$

Many others can be cited.

Noting that labile compounds are more easily attacked by chemical agents than stable ones, Loew[12] has elucidated the action of many poisons. He says: "A systematic toxicological review shows us, among other things, that all compounds acting upon aldehydes and all that easily attack labile amido-groups are poisonous for all kinds of living protoplasm, which fact led me to infer that the lability of the plasma proteids is caused by the presence of aldehyde and amido-groups within the same molecules. * * * The *primum movens* in the living protoplasm must be defined as a mode of motion of labile atoms in the plasma proteins; that is, as a *special case of chemical energy*." According to Loew, the enzymes belong to the dynamically labile compounds.

While the chemical structure of the enzymes is not yet known the researches of Emil Fischer go to show that a knowledge of their constitution is not far beyond our reach. In the fermentation of the sugars Fischer[13] has shown that the enzymes can only "attack those sugars which possess a molecular configuration corresponding to their own,"—that is, they must fit each other "as the key fits its lock." Viewing the enzymes as nucleo-proteid bodies, and as being optically active, he reasoned that *their molecules must have an asymmetric structure.* Their selective action toward $\alpha$ and $\beta$ methyl-glucosides strongly supports this view.

According to Fischer, two methyl-glucosides are formed by the action of hydrochloric acid (HCl) on a solution of *d* glucose in methyl alcohol, and their configuration is given as follows:

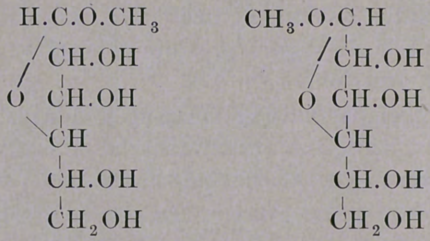

One is called *α*, the other *β*, and their difference is found in the configuration of the one asymmetric carbon atom, yet the enzymes which attack the *α* will not attack the *β*, and *vice versa*. This important discovery sheds a world of light upon the vexed problem of fermentation, and will therefore help to explain many of the obscure phenomena of diseases and of immunity. It will also find a place in the investigation of many of the difficult problems of psysiological chemistry. A very admirable feature of Fischer's hypothesis is its capacity to receive aid from, and give aid to, several other hypotheses—it possesses a wide range of applicability.

In 1892, our esteemed colleague, Professor J. W. McLaughlin,[14] in his book on "Fermentation, Infection and Immunity," elaborated a "Physical Theory," a quotation from which is here presented. After developing the modern conception of complex molecules, Dr. McLaughlin goes on to say: "When we add to this conception of atomic and molecular union, that of atomic vibrations in unvarying periods of time which are distinctive of each kind of atom, and that of etherial wave-motions vibrating in equal periods with the atoms that produce them, the law of 'interference' enables us to understand how atomic wave-motions may be supplemented or antagonized by other atomic wave-motions, and how molecular wave-motions may, likewise, be similarly influenced by other molecular waves; that, in fact, the molecular waves which give a substance its energy will vary with molecular grouping. Now it is in these principles of molecular dynamics, and in chemistry and biology, that, we believe, is to be found the explanation of cell metabolism—constructive and destructive—of fermentation, of infection and immunity." On page 66, he says: "It is only when the molecular vibrations of a ferment, whether this be a living, organized ferment, or a non-living, unorganized ferment, coincide with those of a fermentable substance, that the latter may be disrupted by the former, and fermentation ensue." While these two quotations do not adequately present Dr. McLaughlin's theory, they suggest a connecting link with the physical hypothesis of de Jager.

"Starting with Naegeli's view that fermenting yeast-cells emit vibrations which pass out of the cells and decompose the sugar in the solution surrounding them, de Jager suggests that the enzymes may be regarded not as substances at all, but as the vibrations themselves; that is, as properties of substances rather than material bodies."[15] He compares them to light, electricity, magnetism. That fermentation does not depend upon chemical action of a molecular substance, but that chemical transformations are brought about by physical forces. Maurice Arthus[16] has very ingeniously elaborated the theory of de Jager.

O'Sullivan and Tompson[17] have shown that invertase is capable of inverting more than 100,000 times its own weight of cane sugar without exhausting itself; and Tamman[18] proved that under proper conditions the enzyme is decomposed during its activity with extreme slowness. These reactions find their parallel in the action of nitric oxide in the manufacture of sulphuric acid, and in the action of sulphuric acid in the production of ethyl oxide; and Bredig and von Berneck[19] have recently shown that "one gram-atom (193 grams) of colloidal platinum diffused through seventy million litres of water shows a perceptible action on more than a million times the quantity of hydrogen peroxide."

In these four instances it will be observed that the invertase, nitric oxide, sulphuric acid, and colloidal platinum acted solely in the capacity of catalyzers; that is, they modified the time factor of the reaction—the positive cataylzers accelerating, and the negative catalyzers retarding the velocity of the reactions. Catalyzers, then, serve in the capacity of *liberating impulses.*

That zymohydrolysis is a chemical action finds further support in the recent work of A. Croft Hill[20] on "Reversible Zymohydrolysis." By varying the concentration of mixtures of glucose and maltose he found that the equilibrium point of these two sugars was reached when 85.5% of glucose and 14.5% of maltose were present. Increasing the glucose beyond 85.5% sent the hydrolysis one way, and the reaction reversed when the maltose was increased beyond 14.5%. This is in strict conformity with the law that *"every reaction proceeds to a state of equilibrium, with a definite reaction velocity."*

The phenomena of reversible reactions have been well worked out, and Konowalow's[21] reaction of acetic acid upon pentene:

$$CH_3CO.OH + C_5H_{10} \rightleftarrows CH_3CO.O(C_5H_{11}),$$

has been shown to conform to the requirements of the law of mass-action by Nernst and Hohmann.[22]

Another very important observation made by Bredig and von Berneck[23] is that "relatively minute portions of certain substances are able to inhibit the catalytic action of platinum, and that these are substances which exert a markedly poisonous effect on the living cell and on enzymes. 1/345,000 gram-molecule per litre of hydrogen sulphide already exerts a strongly restraining action. 1/1000 gram-molecule per litre of hydrocyanic acid stops it entirely, and much less is able to retard it greatly. Carbon disulphide, and mercuric chloride show a similar behavior." This again parallels the action of ferments and antiferments.

Were it necessary, many other interesting parallels could be drawn to show the intimate connection between the phenomena of fermentation and the phenomena of chemical action; but this must suffice to authorize the statement that the complex phenomena of fermentation can be best understood when viewed from the pinacle of modern chemical theory:—the Avogadro-van't Hoff Rule, the Phase Rule, Electrolytic Dissociation, and the Doctrine of Energy.

Having shown that chemistry helps us to understand fermentation, let us see what light it is capable of sheding upon

### INFECTION.

The fact that the poisonous action of the bacteria is due to the soluble products formed by the bacteria was established by Panum in 1874. Later, Koch, Chauveau and others succeeded in separating these poisons from the bacteria, and by inoculating animals with them proved that the proliferation of a number of pathogenic organisms in the body was less injurious to the body than the soluble poisons produced by them. Brieger viewed these poisons as organic bases, the so-called ptomaïns; but, subsequently some of them were shown to be either proteid or proteid-like bodies, and many of them acted not unlike digestive ferments. Brieger and Fränkel named the proteid-like bodies toxalbumins. Among the toxins are uncrystallizable poisons the complex chemical structure of which has not yet been made out. The ptomaïns are crystallizable products of bacterial activity somewhat analogous to the vegetable alkaloids. Some possess toxic properties, while others do not. The chemical structure of some of them is well known, but the structure of toxalbumins is a problem for future work.

In his work on "Ptomaines and Leucomaines," Vaughan[24] points out a very interesting chemical relationship between some of the non-poisonous and poisonous members of the cholin group. Starting with trimethyl-ethyl-ammonium hydroxide, by oxidation cholin, neurin, betaïn, and muscarin are derived as follows:

$$\begin{array}{llllll}

CH_3 & & CH_2.OH & CH_2.OH & & CH_2 \\

| & & | & | & & \| \\

CH_2 & +O= & CH_2 & CH_2 & -H_2O= & CH \\

| & & | & | & & | \\

N(CH_3)_3OH & & N(CH_3)_3OH; & N(CH_3)_3OH & & N(CH_3)_3OH;

\end{array}$$

Trimethyl-ethyl-ammonium hydroxide) — (Cholin) (Oxyethyl-trimethyl-ammonium hydroxide) — (Cholin) — (Neurin: Vinyl-trimethyl-ammonium hydroxide)

The structural formulae show the little change that is necessary to convert an innocuous substance into a very poisonous one, and *vice versa.* Cholin is only poisonous in large doses, while very small doses of neurin and muscarin are highly poisonous. Betaïn is not poisonous.

$$\begin{array}{llllll}

CH_2.OH & & CO.OH & CH_2.OH & & CH_2.OH \\

| & & | & | & & | \\

CH_2 & +O_2= & CH_2 & CH_2 & +O= & CH.OH \\

| & & | & | & & | \\

N(CH_3)_3OH & & N(CH_3)_3OH\ ; & N(CH_3)_3OH & & N(CH_3)_3OH\ .

\end{array}$$

Cholin) — (Betaïn: (Trimethyl-glycocoll) — (Muscarin: Dioxyethyl-trimethyl-ammonium-hydroxide)

The structural formulæ show the little change that is necessary to convert an innocuous substance into a very poisonous one, and vice versa. Cholin is only poisonous in large doses, while very small doses of neurin and muscarin are highly poisonous. Betaïn is not poisonous.

Methylguanidin $\left(NH\colon C\begin{array}{l}\diagup NH_2 \\ \diagdown NH.\ (CH_3)\end{array}\right)$, trimethylenediamin $\left(CH_2\begin{array}{l}\diagup CH_2.NH_2 \\ \diagdown CH_2.NH_2\end{array}\right)$, and tyrotoxicon (diazobenzene-potassoxide, $C_6H_5.N_2.OK$) are three other poisonous ptomaïns whose chemical structure is well known. Typhotoxin ($C_7H_{17}NO_2$) is said to be the toxin which gives rise to the typhoid intoxication, and Brieger has been able to separate from tetanus cultures four bases: tetanin ($C_{13}H_{30}N_2O_4$), tetanotoxin ($C_5H_{11}N$), spasmotoxin, and one other unnamed toxin. According to Brieger, each of these is capable of inducing tetanic intoxication. Against this last statement is opposed the further statement that tetanus toxin is a toxalbumin.

Roux, Yersin, and others, succeeded in isolating, seemingly in a state of purity, from the cultures of the Klebs-Loeffler bacillus a toxalbumin, soluble in water, which, when inoculated into a guinea-pig, produced the phenomena characteristic of diphtheria. Prosecuting this line of investigation, these and other investigators have isolated characteristic toxalbumins from cultures of other germs. These toxalbumins have been divided into two principle groups by Brieger and Fränkel, the classification being based upon their solubility. As previously stated, they are proteid-like bodies, highly complex and poisonous. Their further properties may be considered later; but in passing, an idea of their virulence should be given.

"A tetanus toxin has been prepared, of which 0.00005 milligrammes killed a mouse weighing 15 grammes; a man weighing 70 kilogrammes, with the same susceptibility, would be killed by 0.23 milligrammes. This would make the poison 300 times more potent than strychnine."[25]

Closely related to the toxins which arise as products of bacterial activity there is another group of toxic substances which arise in the living animal tissues as the products of either hyper or of retrograde metabolism of the protoplasm, or result from fermentative action. Some of these are proteid-like bodies (toxalbumins), while others are organic bases (leucomaïns) not unlike the vegetable alkaloids.

The chemical structure of many of the leucomaïns is well known; but the same cannot be said of the toxalbumins. The development of their structure must await the unraveling of the proteids—their chemistry seems to flow in channels parallel with the chemistry of the albumins, globulins, albuminates, proteoses and peptones. At least the poisonous principle clings to these products.

The venom of the snake belongs to this class. According to the researches of S. Weir Mitchell,[26] E. T. Reichert,[27] T. R. Fraser[28] and others, snake venom is a very complex mixture, containing, in addition to the poisonous substances, several bodies that are non-poisonous. The poisonous substances are not ferments. Fraser says: "They are substances that produce effects having a direct relationship to the quantity introduced into the body. This quantity in the case of each serpent varies with its size and bodily and mental condition; with the nature of the bite—whether both fangs or only one has been introduced, whether they have penetrated deeply or only scratched the surface; and with other circumstances related to the serpent, such as whether it had recently bitten an animal or not, and thus parted with a portion or retained the whole of the venom stored in the poison glands. * * * The quantity required to produce death being very exactly related to each pound or kilogramme of weight."[29] Fraser found the minimum-lethal dose per kilogramme to be: "For the guinea-pig (of dried venom), 0.00018 gm.; for the frog, 0.0002 gm.; for the rabbit, 0.000245 gm.; for the white rat, 0.00025 gm.; for the cat, somewhat less than 0.005 gm.; and for the grass snake *(Tropedonotus natrix)*, the relatively large dose of 0.03 gm."[30]

It is significant that the toxicity of cobra venom is not the same for all animals. Furthermore, it is exceedingly interesting to find that the experiments carried out by Fraser, *in vitro* and in the animal organism, leave practically no room for doubt that the poisonous action of snake venom and the antagonistic action of antivenin are both chemical. In the unprotected animal snake venom injected beneath the skin or into the blood stream gives immediate evidence of reactions of an endothermic character; but when in the same manner it is introduced into protected animals evidence of exothermic reactions is elicited. When introduced into the stomach of an animal snake venom not only fails to induce symptoms of poisoning, but exhibits a neutralizing action upon inoculated venom, and in the uninoculated animal confers immunity against snake venom. Moreover, the ratio between venom and antivenin is quantitatively brought out in the experiments of Fraser. All this can be shown to be in conformity with well known chemical laws.

Numerous examples which illustrate the chemical nature of the action of toxalbumins might be drawn from various intra- and intercellular protoplasmic bodies found in the vegetable and animal kingdom, but time will not permit their multiplication here. A bountiful supply is to be found in recent biochemical and medical literature, and interested persons are referred to that source.

The specific phenomena of these poisons as exhibited in the human body when toxic quantities are taken will be found in nearly every text-book of modern medicine, so there is no need to repeat them here.

What has been said of the chemistry of fermentation is equally applicable here. Specific illustrations and their enlargement just now would take us beyond the limits of this address; for that reason it is well to pass on to the consideration of the next phase of the subject:

### IMMUNITY.

The problem of immunity is so closely entwined with that of protective inoculation it will be easier to discuss the two conjointly.

In its broadest sense, immunity represents that state of the living organism (animal or vegetable) which enables it to resist the toxic action of substances, whether such substances be introduced from an external source or are developed within the organism. Specific immunity is a state of immunity against a specific substance. This may be *natural,* as when the organism is normally *non-susceptible;* or it may be *artificial* (acquired), as in the case of protection against disease developed by a previous attack of the disease (as in smallpox), or by some other artificial means (vaccination, for instance).

Vexed as the problem is, much enlightenment is to be gained from an investigation of artificial immunity. Recalling the researches of Jenner, and the quotation from Ehrlich relative to the work of Pasteur, at that time it appeared as though artificial immunity was brought about by specific micro-organisms. Opposed to this view the investigations of Toussaint, Chauveau, Salmon and Smith, Roux, C. Fränkel, and others, brought forward evidence to show that artificial immunity could be induced by the "metabolic products" freed from bacteria—accustoming the organism to the specific poison seemed all sufficient. Later it was shown by Hueppe, Gamaleia, and Buchner that the specific toxins found in the culture fluid outside the bacterial cells were not identical with the protective substances found in the germs and their metabolic products.

At this point Hueppe[31] says: It has been "established that: 1, Undergoing the disease; 2, Inoculation with attenuated germs; 3, Inoculation with disease germs which have become wholly impotent; 4, Inoculation with saprophytes; and 5, Inoculation with the metabolic products of the parasite, can all confer immunity; while, 6, Inoculation with the specific poisons effects no immunization." Then followed the experimental proof that completely attenuated bacteria can no longer produce the specific poison. This effectually separates the protective substance and the poison.

The next important advance was the discovery of substances in the blood serum of animals immunized against diphtheria and tetanus that were able to specifically protect other animals against the toxines of these diseases. This discovery was made by Behring, and it at once opened an entirely new and promising field for investigation.

December 3, 1890, in No. 49 of the *Deutsche med. Wochenschrift*, Behring and Kitasato published an article, "Ueber das Zustandekommen der Diphtherie-Immunität und der Tetanus-Immunität bei Thieren," in which the statement is made that: "The blood of tetanus-immunized rabbits possesses the property of destroying tetanus toxin. This is possessed by the extravascular blood, and is the cell free serum." They showed that the blood serum of non-immunized animals did not possess this antagonizing action, and that the prepared serum was of therapeutic value. Ogata and Jasuhara[32] proved that blood serum from an animal naturally immune contained substances which, when injected into mice, conferred upon them the same type of immunity. Tizzoni and Cattani[33] (1891) found that the quantitative protective value of the blood serum of animals naturally immune to tetanus (the dog, for instance) could be greatly increased by repeated injections of gradually increasing amounts of tetanus-toxin; and that such serum possessed decided therapeutic value when inoculated into animals suffering from tetanus. This line of investigation has been greatly extended and enriched by Behring, Roux, Koch, Yersin, Haffkine, Pfeiffer, Buchner, Sanarelli, Ehrlich, and others, and as a result, there is to be found in the open market today a variety of antitoxin sera, such as antidiphtheritic, antitetanic, Marmoreck's antimycotic, antipneumococcic, antibubonic, antirhabic, yellow fever, etc.

March 20, 1896, Professor Thomas R. Fraser, M. D., at the Royal Institution of Great Britain, presented a very important contribution on "Immunisation Against Serpent's Venom, and the Treatment of Snake-bite with Antivenene," in which, for the first time, the quantitative relation between the "toxic" and the "anti" substance is shown. The contribution is rich in splendidly martialed experimental evidence which leads the author to the logical conclusion: that so far as snake venom is concerned, the antidotism of the "antivenene" is not the result of physiological reaction, is not due to phagocytic action, nor to the "resistance of tissues"; but "as I have already pointed out, a chemical theory, implying a reaction between antivenene and venom, which results in a neutralization of the toxic activities of the venom, is entirely compatible with the observed facts."[34]

Another significant fact of chemical importance observed by Fraser is, that in carrying out the immunizing process, "the saturation point of the blood for antivenene is reached before the possible maximum non-fatal dose of venom has been administered." The protective value of venom and "antivenene" when administered by the stomach has already been mentioned.

By this time the use of diphtheria antitoxin as a therapeutic agent in the treatment of diphtheria had become firmly established. The variation in the results obtained caused Ehrlich to search for a quantitative relation between the toxin of diphtheria and the antitoxin of diphtheritic serum. The result of Ehrlich's investigation is to be found in the Croonian lecture delivered by him before the Royal Society, London, March 22, 1900. "By means of test-tube experiments with suspended animal tissues" he brought out some very interesting facts. "The relations were simplest in the case of red blood corpuscles. On them, outside the body, the action of many blood poisons, and of their antitoxines, can be most accurately studied, *e. g.*, the action of ricin, eelserum, snake-poison, tetanus toxine, etc. * * * By means of these test-tube experiments, particularly in the case of ricin, I was able, in the first place, to determine that they yielded an exact quantitative representation of the course of the processes in the living body. * * * It was shown that the action of toxine and antitoxine took place quantitatively as in the animal body. * * * It was proved in the case of certain toxines—notably tetanus toxine —that the action of antitoxines is accentuated or diminished under the influence of the same factors which bring about similar modifications in chemical processes—warmth accelerates, cold retards the reaction, and this proceeds more rapidly in concentrated than in dilute solutions. * * * The knowledge thus gained led easily to the inference that to render toxine innocuous by means of antitoxine was a purely chemical process, in which biological processes had no share."[35]

The distribution of the toxins and the antitoxins in the system is a matter of prime importance, yet not more than a beginning has been made looking toward their localization. That they do possess a selective action has been established by Stokvis, Dönitz, Pfeiffer, Marx, Wasserman and Roux, and these facts throw a great deal of light upon the phenomena of incubation, time reactions, antitoxic action, protective action, serum therapy, etc.

The phenomena of agglutination and lysogenic action, the recent work of Buchner in Germany and Bordet in France on haemolysis, and some experimental work on ionic reactions done in my own laboratory deserve consideration here; but time presses for a summation, and they must be passed without further comment to a future occasion.

From accumulated facts, *acquired immunity* is separable into two distinct types: (The following classification is borrowed from Muir and Ritchie.)[36]

A. Active Immunity—*i. e.,* produced in an animal by an injection, or by a series of injections, of non-lethal doses of an organism or its toxines.

1. *By injection of the living organisms.*
    (*a*) Attenuated in various ways. Examples:—
        (1) By growing in the presence of oxygen, or in a current of air.
        (2) By passing through the tissues of one species of animal (becomes attenuated for another species).
        (3) By growing at abnormal temperatures, etc.
        (4) By growing in the presence of weak antiseptics, or by injecting the latter along with the organism, etc.
    (*b*) In a virulent condition, in non-lethal doses.
2. *By injection of the dead organisms.*
3. *By injection of filtered bacterial cultures, i. e., toxines; or of chemical substances derived from these.*

These methods may also be combined in various ways.

B. Passive Immunity, *i. e.,* produced in one animal by injection of the serum of another animal highly immunized by the methods of A.

1. *By antitoxic serum, i. e.,* the serum of an animal highly immunized against a particular toxine.
2. *By antimicrobic serum, i. e.,* the serum of an animal highly immunized against a particular organism in the living and virulent condition.[36]

The protective value of active immunity extends through a considerable period of time, while that of passive immunity is evanescent.

An adequate explanation of this vast array of facts is yet before us. The explanation in detail cannot be given tonight; that must await another time; but some generalizations must be made.

1. *Pasteur's Theory of Exhaustion* of the pabulum is disproved by the fact of passive immunity.

2. The *Theory of Retention* will have to be greatly modified before it can explain many facts with which it is now in opposition.

3. The *Theory of Acclimatisation or Habituation* has limited application, and like the theory of Adaptation, takes too little cognizance of details.

4. *Metschnikoff's Theory of Phagocytosis* falls before the facts of passive immunity; and

5. The *Humoral Theory* only presents another phase of its own evolution.

6. Buchner's hypothesis, which explains immunity as being due to the reactive changes in the integral cells of the body resulting from the action of chemical products absorbed from the seat of vaccination or inoculation, is strongly supported by experimental evidence; and

7. Ehrlich's[37] side-chain (Seitenkette) theory presents an exceedingly ingenious and interesting explanation of the phenomena of immunity adduced by experiments *in vitro* and *in vivo*..

By elimination the problem may be somewhat simplified. The facts themselves may be roughly divided into two groups: 1. Biological; and 2, Chemical; and the explanations will then be either biological or chemical. In the ultimate analysis: the Biological explanation will rapidly pass from the body as a whole to its respective organs and their respective cells, to the nucleated cells, and finally to the biogen of the nucleus; while the Chemical explanation will describe the cycle that begins with the minutest atomic reaction, passes onward through more and more complex intra- and intermolecular synthetic and analytic changes so long as chemical equilibrium is disturbed; but eventually finds its beginning and its end—cause and effect—in energy potential, energy kinetic, liberating impulse.

That the problem of immunity will be solved is only a question of time. The active research now in progress is rapidly dissipating the unknown; and when the chemical structure of the various animal proteids becomes a known quantity their interaction will be readily seen and the solution of the problem will be an accomplished fact.

The problem is a biochemical one, and biochemists will solve it. Many if not all the phenomena of fermentation, infection and immunity are explainable in terms of modern chemistry, and since modern chemistry is firmly founded on the doctrine of energy we have to consider merely the terms: ENERGY POTENTIAL, ENERGY KINETIC and LIBERATING IMPULSE.

I am conscious of having failed to bring before you a large mass of newly accumulated interesting facts which should be considered in this connection; but the largeness of the subject, together with the enormous accretions annually made to its literature, renders it impossible to present a complete survey of so immense a field of labor in the address of an evening. What has been said is little more than a beginning of what has been done in this line of biochemical research—the promise of its future remains to be told.

Beside the great intellectual gain must be placed the immense practical benefits such investigations have secured for man—as witnessed in the saving of millions of lives of human beings, many times more of the lower animals, and large areas of plant life. They have ever made for the betterment and happiness of man, and for the highest progress of civilization, and so will they continue.

---

*Address at the annual meeting of the Texas Academy of Science, held in the Chemical Theatre of the University of Texas, Austin, Texas, October 26, 1900.

[1a]Presidential Address, British Association, Dover, 1899, by Sir Michael Foster, K. C. B.

[1]Address of welcome on the occasion of the centenary festival of the Royal College of Surgeons of England (July 25-27, 1900), delivered by Sir William MacCormac, Bart., K. C. V. O., etc. (President), and published in *The Lancet*, London, No. 4013, p. 281; also in *Science*, August 17, 1900.

[2]Jenner, Sir William: *Inquiry into the Causes and Effects of the Variolae Vaccinae;* London, May 14, 1796.

[3]Hueppe, Prof. Dr. Ferdinand: *The Principles of Bacteriology*, done into English by Dr. E. O. Jordan (1899), p. 446.

[4]Croonian Lecture: On Immunity with Special reference to Cell Life, by Professor Dr. Paul Ehrlich, Director of the Royal Prussian Institute of Experimental Therapeutics, Frankfort-on-the-Main, read March 22, 1900, before the Royal Society, London, and published in *Proceedings R. S.*, p. 425.

[5]Hueppe, F.: Loc. cit., pp. 225-226.

[6]Buck, A. H., M. D.: A Treatise on Hygiene and Public Health, Vol. I., p. 18.

[7]Hueppe, F.: Loc. cit., pp. 231-232.

[8]*Ibid*, p. 48.

[9]Bokorny and Loew: *Botan. Centralblatt*, 1889 and 1893.

[10]Loew, Oscar: *Science*, June 15, 1900, pp. 932-933; see also *Die Chemische Energie der lebenden Zellen*, von Prof. Dr. Oscar Loew; and *Ein natürliches System der Giftwirkungen*, by the same author, for a full discussion of this subject. (Both books are published by Dr. E. Wolff, Munich.)

[11]Loew: Loc. cit.

[12]*Ibid.*

[13]Fischer, Emil: Einfluss der Configuration auf die Wirkung der Enzyme. *Ber. d. deut. chem. Gesell.*, 1894, **27**, 2985, 3479 and 1895, **28**, 1429.

[14]McLaughlin, J. W., M. D.: *Fermentation, Infection and Immunity*, Austin, Texas, 1892.

[15]Green, J. Reynolds, D. Sc.: *The Soluble Ferments and Fermentation*, Cambridge, 1899, pp. 431-432.

[16]Arthus, Maurice: Natur des Enzymes; *Thèse pour le Doctorat en Médicine*, Paris, 1896.

[17]O'Sullivan and Tompson: Invertase: a Contribution to the History of an Enzyme or Unorganized Ferment. *Jour. Chem. Soc.*, London, 1890, **57**, p. 926.

[18]Tamman, Gustav: Zur Wirkung ungeformter Fermente. *Zeit. f. physik. Chem.*, 1895, **18**, 426-442.

[19]Stokes, H. N.: *Science*, October 12, 1900, p. 555; from Bredig and R. Müller von Berneck, *Zeit. f. physik. Chemic*, **31**, 275 (258-353).

[20]Hill, A. Croft: Reversible Zymohydrolysis, *Jour. Chem. Soc.*, London. 1898, **73**, 634-658.

[21]Konowalow: *Zeitschr. f. physik. Chem.*, 1887, **1**, 63.

[22]Nernst and Hohmann: *Zeitschr. f. physik. Chem.*, 1893, **11**, 352.

[23]Bredig and von Berneck: Loc. cit.

[24]Vaughan and Novy: *Ptomaines and Leucomaines*, 1888, pp. 179-199.

[25]Pearmain and Moore: *Applied Bacteriology*, London, 1898, p. 123.

[26]Mitchell, S. Weir, M. D.: Researches upon the Venoms of Poisonous Serpents. *Contributions to Knowledge*, Smithsonian Institution, 1886.

[27]Reichert, E. T., M. D.: *Ibid.*

[28]Fraser, Prof. Thos. R., M. D.: Immunisation against Serpents' Venom, and the Treatment of Snake-bite with Antivenene. Lecture, Royal Institution, Great Britain, March 20, 1896. *Proceedings* of same, pp. 1-2.

[29]*Ibid*, p. 2.

[30]*Ibid*, p. 8.

[31]Hueppe, F.: Loc. cit., p. 308.

[32]Ogata and Jasuhara: Ueber die Einflüsse einiger Thierblutarten auf Milzbrandbacillen. *Centralbl. für Bacteriol.*, Bd. ix., p. 25.

[33]Tizzoni and Cattani: Ueber die eigenschaften des Tetanus-Antitoxins. *Centralbl. für Bacteriol.*, 1891, Bd. ix., p. 685.

[34]Fraser, T. R.: Loc. cit., p. 18.

[35]Ehrlich: Loc. cit., pp. 428, 429, 431.

[36]Muir and Ritchie: *Manual of Bacteriology*, Edinburgh and London, 1899, p. 462.

[37]Ehrlich: *Klinsches Jahrbuch*, 1897, Bd. vi., Heft 2, S. 309; see also Zur Kenntniss der Antitoxinwirkung, *Fortschritte der Medicin*, 1897, Bd. xv., No. 2; also Croonian Lecture; Loc. cit.

NOTE:

$\longrightarrow$ means: goes into.

$\rightleftharpoons$ means: reversible—goes either way.

